# Prevalence of cultural malpractice during the perinatal period and its determinants among reproductive age women in southwest Ethiopia: A community-based cross-sectional study

**DOI:** 10.3389/fpubh.2023.1064583

**Published:** 2023-03-17

**Authors:** Abinet Tesfaye Diro, Dinaol Abdissa Fufa, Habtamu Geremew

**Affiliations:** ^1^College of Health Science, Oda Bultum University, Chiro, Ethiopia; ^2^School of Public Health, College of Health and Medical Sciences, Mizan-Tepi University, Mizan-Aman, Ethiopia

**Keywords:** prevalence, cultural malpractice, perinatal period, reproductive age women, Ethiopia

## Abstract

**Background:**

Cultural malpractices are accepted cultural norms and socially shared practices that have a negative impact on health. Cultural malpractices vary in type and number in different communities. This study aimed to determine the prevalence of cultural malpractice during the perinatal period and identify its predictors among reproductive-age women in rural communities of southwestern Ethiopia.

**Methods:**

A community-based cross-sectional study was conducted from May 5 to 31, 2019 in Semen Bench district, southwestern Ethiopia; among reproductive-age women who had experienced at least one prior delivery. A systematic random sampling technique was employed to select 422 women for the interview. After collection, the data were entered into EpiData and exported to STATA-14 for further analysis. Descriptive analyses were performed and presented in texts and tables. Besides, binary and multivariable logistic regressions were computed to identify determinants of cultural malpractice.

**Result:**

A total of 414 women completed the survey, resulting in a response rate of 98%. We found that 26.33% (95% CI: 22.15, 30.85%) had food taboos during pregnancy, 31.88% (95% CI: 27.42, 36.61%) delivered their last child at home, and 33.82% (95% CI: 29.27, 38.6%) practiced pre-lacteal feeding. Lack of formal education (AOR: 11.22, 95% CI: 6.24, 20.15), lack of ANC follow-up (AOR: 10.82, 95% CI: 5.46, 21.42), rural residence (AOR: 6.23, 95% CI: 2.18, 17.78), and avoiding colostrum (AOR: 21.94, 95% CI: 9.73, 49.48) were significantly associated with cultural malpractice during the perinatal period.

**Conclusion:**

The prevalence of cultural malpractice is notably high in the study area. Hence, community-based measures including expansion of education and promotion of maternal health services are important to reduce cultural malpractice during the perinatal period.

## Introduction

Different communities have socially shared practices that are deeply rooted in their culture and have an important association with their health (1). Cultural malpractices are accepted cultural norms and socially shared practices that have a negative impact on health (2, 3). The perinatal period, which includes pregnancy, delivery, and the post-natal period, is accompanied by various cultural malpractices that substantially affect the health of the mother and her children (4, 5).

Globally, several types of cultural malpractices have been reported (2, 6, 7). Ethiopia, as a multi-ethnic nation, is one of the countries where assorted cultural malpractices particularly during pregnancy, delivery, and post-natal period are practiced; among these, home delivery, pre-lacteal feeding, food prohibition, abdominal massage, and avoiding of colostrum were commonly documented (5, 8–11). Bench Maji Zone is also a home for such malpractices where home delivery, food prohibition, pre-lacteal feeding, and abdominal massage are commonly exercised (8, 12). These malpractices were found to have a significant association with residence, educational status, antenatal care (ANC) follow-up, gravidity, and distance from the health facility (5, 10, 13–15).

Cultural malpractices not only hinder women from utilizing maternal, reproductive, and child health services, but they also have a direct impact on the wellbeing and survival of both the mother and her offspring (2, 7, 16). Previously, studies have concentrated on the biomedical causes and management of maternal death; yet, they ignored cultural and traditional practices that can deter maternal morbidity and mortality (6, 17).

Maternal mortality is extremely high worldwide, accounting for 295,000 pregnancy-related deaths in 2017; almost all (94%) of these deaths occurred in resource-constrained settings (18, 19). Ethiopia is one of the nations where a higher number of maternal deaths are reported; a recent demographic and health survey estimated that there were around 412 maternal deaths per 100,000 live births (20). Various strategies, like improving access to reproductive health care and maternal death surveillance and response systems, have been implemented to reduce maternal mortality in Ethiopia; however, it persists as a major public health concern (21, 22). Cultural malpractices are among the important contributors to the high maternal mortality rate (23). It is estimated that about 5–15% of maternal deaths are due to cultural malpractice (6, 10, 24).

Cultural malpractices vary in type and number across different communities (8, 9, 25, 26). Thus, comprehensive evidence about the magnitude and determinant factors of cultural malpractices, especially during the perinatal period across different societies is crucial to reduce maternal mortality. Hence, this study was conducted to determine the prevalence of cultural malpractice during the perinatal period and identify associated factors among reproductive-age women in rural communities of Semen Bench district, southwestern Ethiopia.

## Materials and methods

### Study area, design, and period

A community-based, cross-sectional study was conducted from May 5 to 31, 2019 in the Semen Bench district. It is one of the ten decentralized districts in the Bench Maji zone, Southern Nation, Nationalities, and Peoples Region (SNNP) of Ethiopia. The district is composed of thirty-one Kebeles (the smallest administrative unit in Ethiopia), of which twenty-eight were rural. The estimated total population of the district was 159,480; of which 87,748 (51.26%) were females; the majority of the population in the district (152,973) are rural residents, with agriculture serving as their main economic source (27). There are four health centers and thirty-one health posts that provide maternal and child health services in the district.

### Study participants

#### Source population

All reproductive-age women in the Semen Bench district who had experienced at least one prior delivery.

#### Study population

All reproductive-age women in randomly selected kebeles of the Semen Bench district who had experienced at least one prior delivery.

#### Inclusion criteria

All women who had experienced at least one delivery and were available during the period of data collection were included in the study.

#### Exclusion criteria

Women who were seriously ill and/or had difficulties to communicate were excluded.

### Sample size determination and sampling procedure

The sample size was determined based on the single population proportion formula considering a 95% confidence interval, 5% margin of error, and cultural malpractice proportion of 50.9% from a previous study (28).


$$\begin{array}{l} \text{Thus},\;n=Z=\frac{\left(\frac{z\;\alpha }{2}\right)2}{d2}p\left(1-p\right) \end{array}$$

where,

*n* = is the desired sample size.

*Z*α*/*2 = the value of standard normal distribution corresponding to the significance level at α of 5%, which is 1.96.

*P* = is the proportion of cultural malpractice.

Therefore,


$$\begin{array}{l} n=\frac{\;\left(\;1.96\right)2}{\left(0.05\right)2}0.51\left(1-0.51\right)=\;\frac{\left(3.8416\right)}{0.0025}0.509\left(1-0.509\right)\;=384 \end{array}$$

Adding 10% non-response; *n* = 384 + (10^*^384/100) = 422.

Therefore, the final sample size for the study was 422 women of reproductive age.

**Figure 1:**
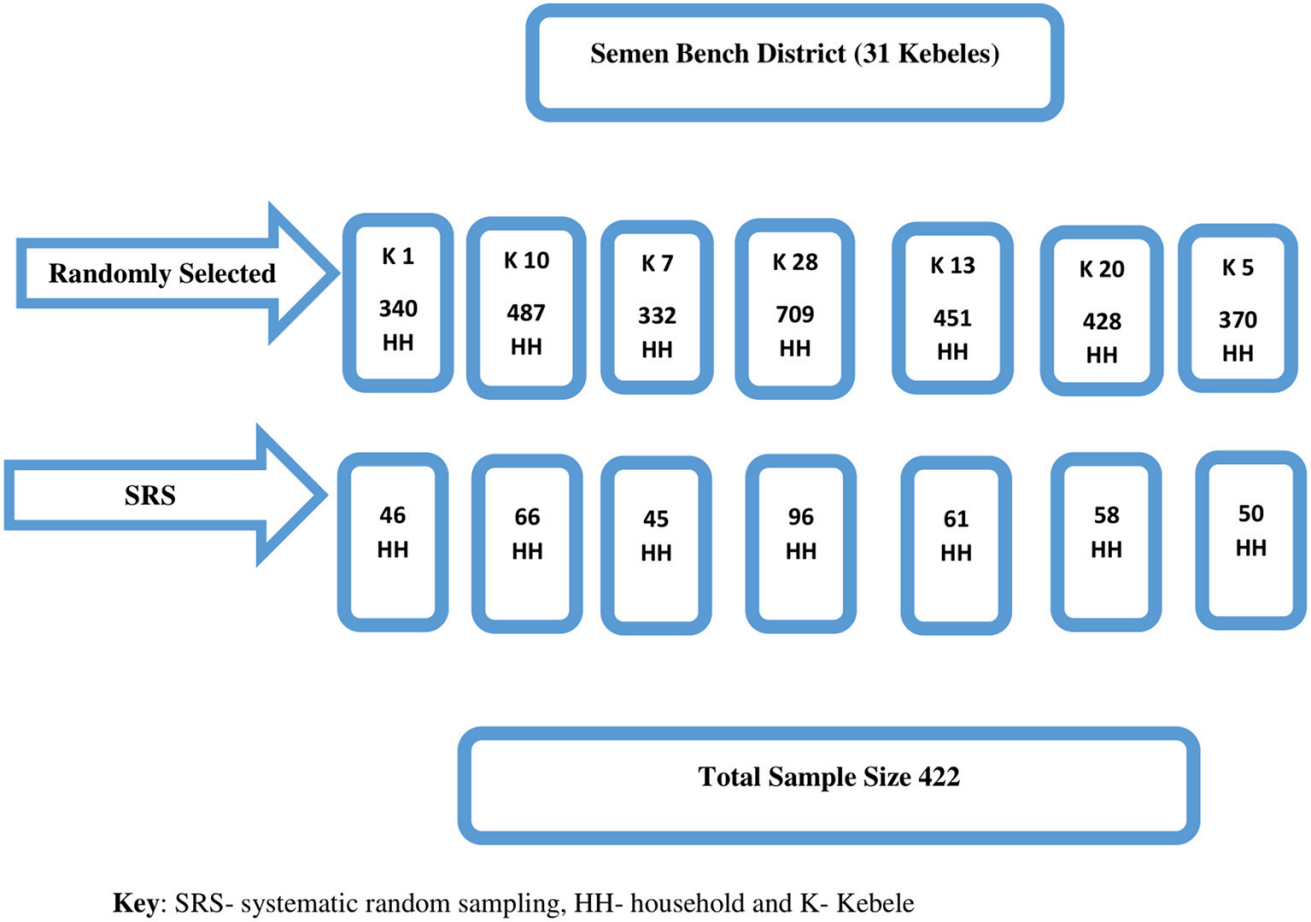
Schematic presentation of the sampling procedure to assess cultural malpractice during the perinatal period in southwest Ethiopia.

Out of the thirty-one kebeles in the district, seven kebeles (one urban and six rural) were randomly selected. The study participants were recruited using systematic random sampling by considering household lists of randomly selected kebeles as sampling frames. For a household without eligible women, the next household was considered. Conversely, for households with more than one eligible woman, one of the women was selected randomly (Figure 1).

### Data collection tool, procedure, and quality control

A structured questionnaire (see Supplementary material), that was adapted from different literature was used (10, 11, 28). A face-to-face interview was employed to collect the data through house to house survey. The data were collected by seven midwives and they were supervised by two public health professionals. To assure the quality of the study, 2 days training was given to data collectors and supervisors about the objectives of the study and techniques of data collection. The tool was also pretested on 5% of the calculated sample size in one of the kebeles not included in this study. Furthermore, the collected data were checked every day for quality, completeness, and consistency.

### Operational definitions

**Perinatal period**: denotes the period during pregnancy, delivery, and postnatal period (5).

**Cultural malpractice**: accepted cultural practices that have a negative impact on health (5, 10).

**Food taboo**: avoiding certain foods during some occasions, like pregnancy, due to cultural beliefs (3, 10).

**Pre-lacteal feeding**: feeding the neonate something other than breast milk in the first 3 days after birth (29).

### Data processing and analysis

The collected data were entered into EpiData and exported to STATA version 14 for cleaning, categorizing, and further statistical analysis. Consequently, descriptive and inferential statistics were computed using the software. The bi-variable and multi-variable logistic regressions were employed to identify factors associated with cultural malpractice after checking for assumptions and assessing multicollinearity. Variables with a *P*-value < 0.25 during bi-variable analyses were chosen for subsequent multi-variable logistic regression. In the multi-variable analysis, significant statistical association was declared at a *P*-value < 0.05. Besides, the Hosmer-Lemeshow goodness-of-fit test and classification table were used to verify the fitness of the final models.

## Results

### Socio-demographic characteristics of participants

From the total calculated sample size of 422 reproductive-age women, 414 women completed the survey resulting in a response rate of 98%. Almost two out of every five women had no formal education. More than two-thirds of the respondents were rural dwellers. Most (84.3%) of the women were married, and the rest were either widowed (11.8%) or divorced (3.9%). More than half of the participants were housewives (Table 1).

**Table 1:** Socio-demographic characteristics of study participants to assess cultural malpractice during the perinatal period in southwest Ethiopia.

**Variable**	**Category**	**Frequency**	**Percentage**
Age	15–24	108	26.1
	25–34	159	38.4
	>35	147	35.5
Residence	Urban	95	22.9
	Rural	319	77.1
Marital status	Married	349	84.3
	Widowed	49	11.8
	Divorced	16	3.9
Educational status	No formal education	177	42.7
	Primary and above	237	57.3
Occupation	House wife	246	59.4
	Merchant	78	18.9
	Governmental employee	58	14.0
	Others^a^	32	7.7
Ethnicity	Bench	242	58.5
	Amhara	75	18.1
	Oromo	44	10.6
	Kefa	37	8.9
	Others^b^	16	3.9
Religion	Orthodox	179	43.2
	Protestant	161	38.9
	Muslim	74	17.9

Others^a^, students and private employees; Others^b^, Sheka, Sheko and Dize.

### Obstetrical characteristics of participants

Among the participants, 178 had an antenatal care (ANC) follow-up. About one-third (35.3%) of the women had an illness during their last pregnancy. Most of the home deliveries were attended by families, and about one-fourth of women avoided feeding colostrum (Table 2).

**Table 2:** Obstetrical characteristics of study participants to assess cultural malpractice during the perinatal period in southwest Ethiopia.

**Variable**	**Category**	**Frequency**	**Percentage**
Gravida	1–2	158	38.2
	3–4	159	38.4
	>5	97	23.4
Illness during pregnancy	Yes	146	35.3
	No	268	64.7
ANC follow-up	Yes	178	43.0
	No	236	57.0
Who attended the delivery at home	Family	62	47.0
	Neighbors	33	25.0
	UTTBA	25	18.9
	TTBA	12	9.1
Colostrum feeding	Yes	304	73.4
	No	110	26.6

UTTBA, un-trained traditional birth attendants; TTBA, trained traditional birth attendants.

### Prevalence of cultural malpractice

**Figure 2:**
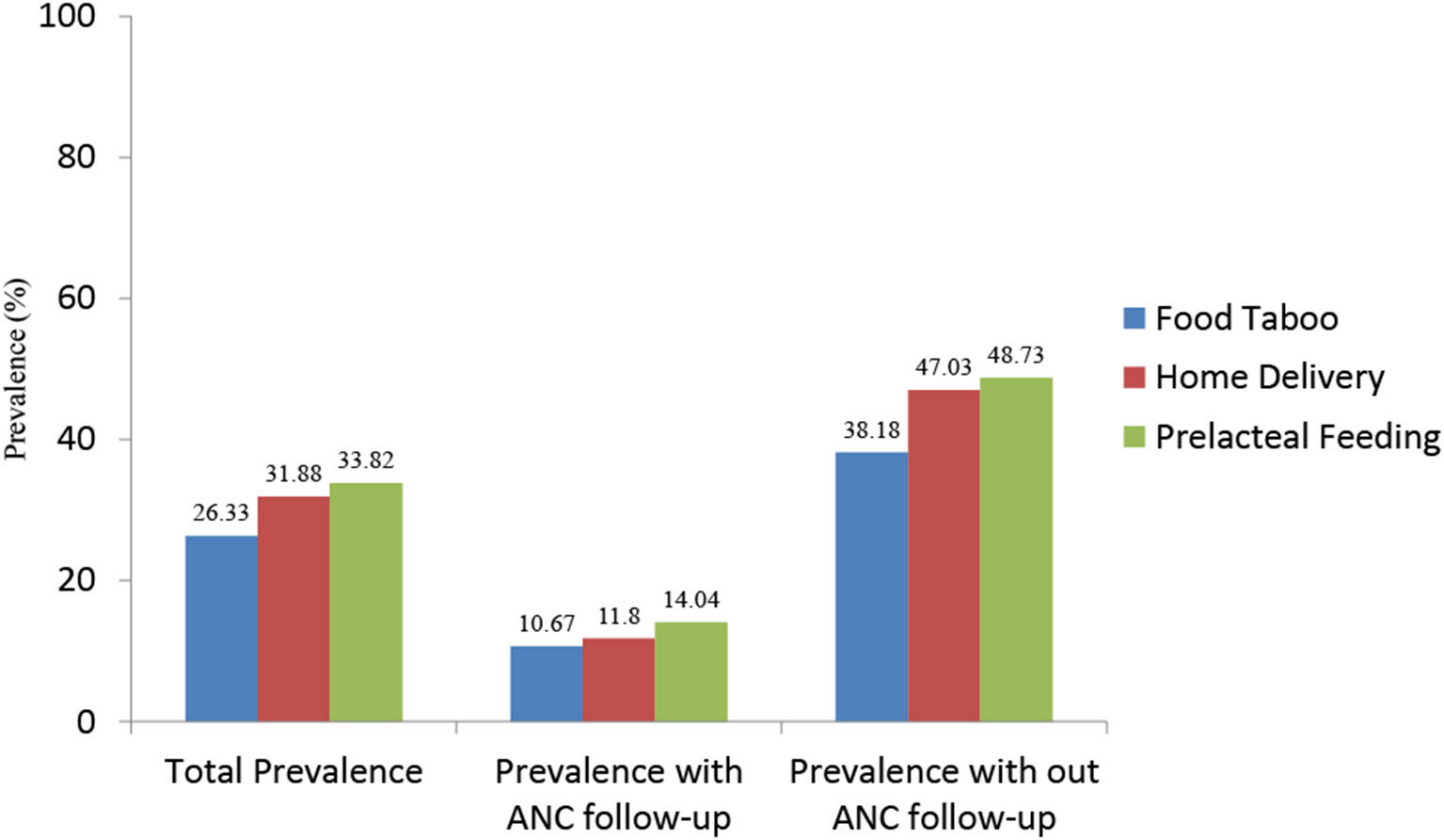
Prevalence of cultural malpractice during the perinatal period, by ANC follow-up status of women in southwest Ethiopia.

Out of 414 women who completed this survey; 26.33% (95% CI: 22.15, 30.85%) had food restrictions during pregnancy, 31.88% (95% CI: 27.42, 36.61%) delivered their last child at home, and 33.82% (95% CI: 29.27, 38.6%) practiced pre-lacteal feeding on their newborn. Other cultural malpractices like abdominal massage, uterine massage, using an unclean blade to cut the umbilical cord, shaking of the abdomen or uterus, applying cow dung or butter on the umbilical stump, late initiation of breastfeeding, and avoiding the colostrum were also reported (Figure 2).

### Factors associated with cultural malpractice

Various factors were found to have a statistically significant association with the most commonly reported cultural malpractices (food taboo, home delivery, and pre-lacteal feeding).

Factors associated with food taboo: residence, educational status, and ANC follow-up were significantly associated with food taboo during pregnancy. Accordingly, rural dwellers were 2.25 (95% CI: 1.15, 4.38) times at higher risk of food taboo than urban residents. Similarly, women who did not attend formal education were having a 2.25 (95% CI: 1.15, 4.38) times higher risk of food taboo as compared to women who attended primary and above education. The odds of food taboo were 4.56 (95% CI: 2.61, 7.97) times higher among women who did not have ANC follow-up than among women who had (Table 3).

**Table 3:** Factors associated with food taboo during pregnancy among reproductive age women in southwest Ethiopia.

**Variables**	**Category**	**Food Taboo**		**CR**	**AOR (95% CI)**
		**No**	**Yes**		
Age	15–24	98	15	0.31	0.58 (0.27, 1.24)
	25–34	111	46	0.83	1.28 (0.72, 2.280
	>35	96	48	Ref	Ref
Residence	Urban	82	13	Ref	Ref
	Rural	223	96	2.72	2.25 (1.15, 4.38)^*^
Educational status	No formal education	113	64	2.42	2.03 (1.18, 3.51)^*^
	Primary and above	192	45	Ref	Ref
ANC follow-up	No	146	90	5.16	4.56 (2.61, 7.97)^*^
	Yes	159	19	Ref	Ref

AOR, adjusted odds ratio; CI, confidence interval; COR, crude odds ratio; ^*^Significant at *P*-value < 0.05.

Factors associated with home delivery: marital status, educational status, and ANC follow-up significantly determined home delivery practice. Widowed and divorced women had a 5.97 (95% CI: 2.79, 12.78) times higher risk of home delivery than married ones. Women with no formal education were 11.22 (95% CI: 6.24, 20.15) times more likely to give birth at home as compared to women who attended primary and above education. Likewise, women who didn't have ANC follow-up were having a 10.82 (95% CI: 5.46, 21.42) times higher risk of home delivery than women who had ANC follow-up (Table 4).

**Table 4:** Factors associated with home delivery among reproductive age women in southwest Ethiopia.

**Variables**	**Category**	**Place of delivery**		**CR**	**AOR (95% CI)**
		**Health institution**	**Home**		
Residence	Urban	77	18	Ref	Ref
	Rural	205	114	2.38	1.82 (0.88, 3.76)
Marital status	Married	262	87	Ref	Ref
	Widowed/Divorced	20	45	6.78	5.97 (2.79, 12.78)^*^
Educational status	No formal education	73	104	10.63	11.22 (6.24, 20.15)^*^
	Primary and above	209	28	Ref	Ref
ANC follow-up	No	125	111	6.64	10.82 (5.46, 21.42)^*^
	Yes	157	21	Ref	Ref
Illness during pregnancy	Yes	109	37	0.62	1.45 (0.8, 2.62)
	No	173	95	Ref	Ref

AOR, adjusted odds ratio; CI, confidence interval; COR, crude odds ratio; ^*^Significant at *P*-value < 0.05.

Factors associated with pre-lacteal feeding: rural dwellers were 6.23 (95% CI: 2.18, 17.78) times more likely to practice pre-lacteal feeding than urban residents. Respondents who were Amhara in their ethnicity were 4.94 (95% CI: 2.09, 11.66) times more likely to practice pre-lacteal feeding than Bench ethnic groups. Similarly, women who didn't attend formal education had a 6.30 (95% CI: 2.67, 14.87) times higher risk of pre-lacteal feeding as compared to women who attended primary and above education. Women who had five or more gravida were 7.17 (95%: 2.95, 17.44) times more likely to practice pre-lacteal feeding than women who had fewer gravida. Likewise, women who didn't have ANC follow-up were having 6.73 (95% CI: 3.1, 14.61) times higher risk of pre-lacteal feeding as compared to women who had ANC follow-up. Women who avoided feeding colostrum to their newborns were 21.94 (95% CI: 9.73, 49.48) times more likely to practice pre-lacteal feeding than their counterparts (Table 5).

**Table 5:** Factors associated with pre-lacteal feeding among reproductive age women in southwest Ethiopia.

**Variables**	**Category**	**Pre-lacteal feeding**		**CR**	**AOR (95% CI)**
		**No**	**Yes**		
Residence	Urban	80	15	Ref	Ref
	Rural	194	125	3.43	6.23 (2.18, 17.78)^*^
Ethnicity	Bench	181	61	Ref	Ref
	Amhara	36	39	3.21	4.94 (2.09, 11.66)^*^
	Oromo	24	20	2.47	2.56 (0.83, 7.87)
	Others^@^	33	20	1.78	0.69 (0.25, 1.9)
Educational status	No formal education	74	103	7.52	6.30 (2.67, 14.87)^*^
	Primary and above	200	37	Ref	Ref
Gravida	1–2	137	21	0.38	1.36 (0.56, 3.32)
	3–4	113	46	Ref	Ref
	>5	24	73	7.47	7.17 (2.95, 17.44)^*^
ANC follow-up	No	121	115	5.82	6.73 (3.1, 14.61)^*^
	Yes	153	25	Ref	Ref
Colostrum feeding	Yes	254	50	Ref	Ref
	No	20	90	22.86	21.94 (9.73, 49.48)^*^

AOR, adjusted odds ratio; CI, confidence interval; COR, crude odds ratio; ^@^Kefa, Sheka, Sheko, and Dize; ^*^Significant at *P*-value < 0.05.

## Discussion

This study was conducted to estimate the prevalence of cultural malpractice during the perinatal period and identify factors associated with it among reproductive-age women in rural communities of southwest Ethiopia. Consequently, the prevalence of food taboo during pregnancy, home delivery, and pre-lacteal feeding were 26.33, 31.88, and 33.82% respectively. Besides; residence, marital status, educational status, ANC follow-up, gravidity, and avoidance of colostrum were found to have a significant association with cultural malpractices.

We found that the prevalence of food taboo during pregnancy was 26.33% (95% CI: 22.15, 30.85%). This finding was higher than previous reports from different parts of Ethiopia (10, 11, 28, 30). The possible explanation could be due to differences in residence and period of study. Previous studies were conducted among urban dwellers whereas the current study mainly included rural women. Besides, this study estimated food taboos during pregnancy, whereas the previous studies investigated food taboos during delivery and the postnatal period. On the other hand, this finding was lower than previous researches from Ethiopia (5, 31), and Nepal (25). This could be partly because of socio-cultural variations.

Our study also found that the prevalence of home delivery was 31.88% (95% CI: 27.42, 36.61%). This result was higher than previous reports in Ethiopia (13, 15, 32). This might be due to the difference in the study setting; the previous studies were institutional based while our study was community-based. However, our finding was lower than studies conducted in the Afar and Benishangul Gumuz Regions of Ethiopia (33–36). The possible explanation could be due to the poor infrastructure in the underserved regions of the country, thus leading to poor access to health care.

Furthermore, this analysis indicated that the prevalence of pre-lacteal feeding was 33.82% (95% CI: 29.27, 38.6%). This finding is lower than previous researches conducted in the same country (37–39), this might be due to the difference in time of study; these studies were conducted in earlier times when access and quality of health care were relatively suboptimal. In contrast, our finding was higher than previous reports from various parts of Ethiopia (40–43). This might be attributed to the socio-cultural variation across the country.

Moreover, we also identified determinants of cultural malpractice among reproductive-age women in southwest Ethiopia. Accordingly, ANC follow-up and educational status had a consistent association with the identified cultural malpractices. In line with previous studies, women who didn't have ANC follow-up had a higher risk of food taboo, home delivery, and pre-lacteal feeding (5, 10, 34). This might be attributed to the effect of counseling and client education during ANC (43, 44). Similarly, women with no formal education were more likely to practice food taboos, home delivery, and pre-lacteal feeding than women with primary and above education. This association was documented elsewhere (5, 10, 13, 15, 29), and could be related to the lack of awareness about the harmful effect of cultural malpractices on the health of the mother and her offspring among women without formal education.

The results of this study indicated that residence is significantly associated with food taboos during pregnancy and pre-lacteal feeding. Rural residents were having a higher risk of food taboo and pre-lacteal feeding than urban women. This finding was also reported by previous studies (5, 10, 45). One possible explanation is that rural residents have limited access to health care and are less exposed to health information (42, 46).

Our study also identified that marital status is significantly associated with home delivery. Divorced and/or widowed women had a higher risk of home delivery as compared to married women. This association was also supported by previous reports (47, 48), and might be due to the enabling effect of partner support on improving maternal and reproductive health care service utilization (49, 50).

This study revealed that respondents who were Amhara in their ethnicity were more likely to practice pre-lacteal feeding than Bench ethnic groups. This could be partly due to the difference in socio-cultural attributes between the two ethnic groups. Consistent with previous studies, women who have had five or more gravidity were more likely to practice pre-lacteal feeding than their counterparts (5, 26). This could be due to the higher prevalence of cultural beliefs among older women. Lastly, we found that avoiding the colostrum was strongly associated with pre-lacteal feeding. This association was documented elsewhere (29, 43, 51) and might be due to the misconception among mothers that colostrum is non-nutritious and causes diarrhea (52).

## Limitations

The findings of this study should be interpreted considering the following limitations. Firstly, as we investigated events that occurred in the past, recall bias could not be ruled out. Secondly, the cross-sectional nature of the study makes it difficult to establish a temporal association.

## Conclusion

The prevalence of cultural malpractice is notably high in the study area. Educational status, ANC follow-up, residence, marital status, gravidity, and avoiding colostrum had a significant association with cultural malpractices during the perinatal period. Hence, community-based measures, including the expansion of education and promotion of maternal health services are important to reduce cultural malpractice during the perinatal period.

## Data availability statement

The raw data supporting the conclusions of this article will be made available by the authors, without undue reservation.

## Ethics statement

Ethical clearance was obtained from the College of Health Science, Mizan-Tepi University Ethical Review Board. Considering the non-invasive nature of data collection procedures, verbal consent was obtained from each woman who participated in the survey after a detailed explanation of the purpose of the research and the right to withdraw from the study at any time. For women who practiced cultural malpractice, detailed descriptions of possible health consequences and appropriate alternative measures were discussed by data collectors. Besides, confidentiality was assured by not recording personal identifiers and using the data only for the purpose of this study.

## Author contributions

ATD conceived the study. ATD, DAF, and HG designed the study, supervised data collection, analyzed the data, wrote the first draft, and critically reviewed the manuscript. All authors have read and approved the final manuscript.

## References

[1] Gurung RA. Cultural influences on health. Cross Cult Psychol Contemp Themes Perspect. (2019) 15:451–66. 10.1002/9781119519348.ch21

[2] Wells Y-O Dietsch E. Childbearing traditions of Indian women at home and abroad: an integrative literature review. Women Birth. (2014) 27:e1–6. 10.1016/j.wombi.2014.08.006 25257377

[3] Ebabu A Muhammed M. Traditional practice affecting maternal health in pastoralist community of afar region, Ethiopia: a facility-based cross-sectional study. J Midwifery Reprod Health. (2021) 9:2817–27. 10.22038/JMRH.2021.46790.1575

[4] Chand S. Cultural beliefs and traditional rituals about child birth practice in rural, Nepal. MOJ Public Health. (2016) 5:00106. 10.15406/mojph.2016.04.00106

[5] Abebe H Beyene GA Mulat BS. Harmful cultural practices during perinatal period and associated factors among women of childbearing age in Southern Ethiopia: community based cross-sectional study. PLoS ONE. (2021) 16:e0254095. 10.1371/journal.pone.0254095 34214133 PMC8253409

[6] Marabele PM Maputle MS Ramathuba DU Netshikweta L. Cultural factors contributing to maternal mortality rate in rural villages of Limpopo Province, South Africa. Int J Women's Health. (2020) 12:691–9. 10.2147/IJWH.S231514 32943943 PMC7468369

[7] Ugboma H Akani C. Abdominal massage: another cause of maternal mortality. Nigerian J Med J Natl Assoc Resid Doctors Nigeria. (2004) 13:259–62. 15532228

[8] Solomon N Tesfaye M. Traditional practices during pregnancy and childbirth among mothers in Shey Bench District, South West Ethiopia. SAGE Open Med. (2022) 10:20503121221098139. 10.1177/20503121221098139

[9] Tsegaye D Tamiru D Belachew T. Food-related taboos and misconceptions during pregnancy among rural communities of Illu Aba Bor zone, Southwest Ethiopia. A community based qualitative cross-sectional study. BMC Pregn Childbirth. (2021) 21:309. 10.1186/s12884-021-03778-6 33865339 PMC8052673

[10] Melesse MF Bitewa YB Dessie KN Wondim DB Bereka TM. Cultural malpractices during labor/delivery and associated factors among women who had at least one history of delivery in selected Zones of Amhara region, North West Ethiopia: community based cross-sectional study. BMC Pregn Childbirth. (2021) 21:504. 10.1186/s12884-021-03971-7 34253187 PMC8273571

[11] Tola TN Tadesse AH. Cultural malpractices during pregnancy, child birth and postnatal period among women of child bearing age in Limmu Genet Town, Southwest Ethiopia. Sci J Public Health. (2015) 3:752–6. 10.11648/j.sjph.20150305.32

[12] Henok A Takele E. Assessment of barriers to reproductive health service utilization among Bench Maji Zone Pastoralist Communities. Ethiop J Health Sci. (2017) 27:523–30. 10.4314/ejhs.v27i5.11 29217958 PMC5615014

[13] Hailu D Tadele H Tadesse BT Alemayehu A Abuka T Woldegebriel F. Home delivery practice and its predictors in South Ethiopia. PLoS ONE. (2021) 16:e0254696. 10.1371/journal.pone.0254696 34370742 PMC8351986

[14] Ayenew AA Nigussie AA Zewdu BF. Childbirth at home and associated factors in Ethiopia: a systematic review and meta-analysis. Arch Public Health Arch Belges de Sante Publique. (2021) 79:48–48. 10.1186/s13690-021-00569-5 33849638 PMC8042927

[15] Siyoum M Astatkie A Mekonnen S Bekele G Taye K Tenaw Z. Home birth and its determinants among antenatal care-booked women in public hospitals in Wolayta Zone, southern Ethiopia. PLoS ONE. (2018) 13:e0203609. 10.1371/journal.pone.0203609 30192861 PMC6128615

[16] Turner C Pol S Suon K Neou L Day NP Parker M. Beliefs and practices during pregnancy, post-partum and in the first days of an infant's life in rural Cambodia. BMC Pregn Childbirth. (2017) 17:1–8. 10.1186/s12884-017-1305-9 28403813 PMC5389162

[17] Geller SE Goudar SS Adams MG Naik VA Patel A Bellad MB. Factors associated with acute postpartum hemorrhage in low-risk women delivering in rural India. Int J Gynaecol Obstet Off Organ Int Feder Gynaecol Obstet. (2008) 101:94–9. 10.1016/j.ijgo.2007.08.025 18291401 PMC3711742

[18] WHO. Maternal Mortality: Evidence Brief. Geneva: World Health Organization (2019).

[19] WHO UNFPA World Bank Group and the United Nations Population Division. Trends in maternal mortality 2000–2017: estimates by WHO, UNICEF. In: UNFPA, World Bank Group and the United Nations Population Division. Geneva: WHO (2019).

[20] Central Statistical Agency (CSA) [Ethiopia] and ICF. Ethiopia Demographic and Health Survey 2016. Addis Ababa, Ethiopia, and Rockville, Maryland: CSA and ICF (2016).

[21] Abebe B Busza J Hadush A Usmael A Zeleke AB Sita S. ‘We identify, discuss, act and promise to prevent similar deaths': a qualitative study of Ethiopia's Maternal Death Surveillance and Response system. BMJ Global Health. (2017) 2:e000199. 10.1136/bmjgh-2016-000199 28589016 PMC5435261

[22] Yaya Y Data T Lindtjørn B. Maternal mortality in rural south Ethiopia: outcomes of community-based birth registration by health extension workers. PLoS ONE. (2015) 10:e0119321. 10.1371/journal.pone.0119321 25799229 PMC4370399

[23] Evans EC A. review of cultural influence on maternal mortality in the developing world. Midwifery. (2013) 29:490–6. 10.1016/j.midw.2012.04.002 23149237

[24] Central Statistical Agency [Ethiopia] and ICF International. Ethiopia Demographic and Health Survey 2011. Addis Ababa, Ethiopia and Calverton, Maryland: Central Statistical Agency and ICF International (2012).

[25] Ramulondi M de Wet H Ntuli NR. Traditional food taboos and practices during pregnancy, postpartum recovery, and infant care of Zulu women in northern KwaZulu-Natal. J Ethnobiol Ethnomed. (2021) 17:1–19. 10.1186/s13002-021-00451-2 33743760 PMC7981893

[26] Igwenyi PI Nwankwo O Nwafor JN Eunice AN Aleke CO Obande-Ogbuinya EN. Demographic predictors of cultural practices regarding female genital mutilation among married women in Ebonyi State, Nigeria. J Adv Med Med Res. (2021) 33:23–31. 10.9734/jammr/2021/v33i830882

[27] Ethiopian Census Current Year Projection - SNNP - Bench Maji - Semien Bench Navigate the Semien Bench Woreda Population Projection. (2021). Available online at: https://www.qotera.org/en-US/snnp/bench-maji/semien-bench/ (accessed March 19, 2021).

[28] Gedamu H Tsegaw A Debebe E. The prevalence of traditional malpractice during pregnancy, child birth, and postnatal period among women of childbearing age in Meshenti Town, 2016. Int J Reprod Med. (2018) 2018:5945060. 10.1155/2018/5945060 29568739 PMC5820650

[29] Sorrie MB Amaje E Gebremeskel F. Pre-lacteal feeding practices and associated factors among mothers of children aged less than 12 months in Jinka Town, South Ethiopia, 2018/19. PLoS ONE. (2020) 15:e0240583. 10.1371/journal.pone.0240583 33048981 PMC7553318

[30] Tela FG Gebremariam LW Beyene SA. Food taboos and related misperceptions during pregnancy in Mekelle city, Tigray, Northern Ethiopia. PLoS ONE. (2020) 15:e0239451. 10.1371/journal.pone.0239451 33048926 PMC7553351

[31] Zepro NB. Food taboos and misconceptions among pregnant women of Shashemene District, Ethiopia, 2012. Sci J Public Health. (2015) 3:410–6. 10.11648/j.sjph.20150303.27

[32] Yoseph M Abebe SM Mekonnen FA Sisay M Gonete KA. Institutional delivery services utilization and its determinant factors among women who gave birth in the past 24 months in Southwest Ethiopia. BMC Health Serv Res. (2020) 20:1–10. 10.1186/s12913-020-05121-9 32228558 PMC7106731

[33] Mitikie KA Wassie GT Beyene MB. Institutional delivery services utilization and associated factors among mothers who gave birth in the last year in Mandura district, Northwest Ethiopia. PLoS ONE. (2020) 15:e0243466. 10.1371/journal.pone.0243466 33326426 PMC7743934

[34] Berhe R Nigusie A. Magnitude of home delivery and associated factors among child bearing age mothers in Sherkole District, Benishangul Gumuz regional state-Western-Ethiopia. BMC Public Health. (2020) 20:1–7. 10.1186/s12889-020-08919-8 32460736 PMC7251823

[35] Mekonnen MG Yalew KN Umer JY Melese M. Determinants of delivery practices among Afar pastoralists of Ethiopia. Pan Afr Med J. (2012) 13:17. 23467618 PMC3587020

[36] Assefa L Alemayehu M Debie A. Magnitude of institutional delivery service utilization and associated factors among women in pastoral community of Awash Fentale district Afar Regional State, Ethiopia. BMC Res Notes. (2018) 11:1–6. 10.1186/s13104-018-3261-5 29499736 PMC5833063

[37] Bekele Y Mengistie B Mesfine F. Prelacteal feeding practice and associated factors among mothers attending immunization clinic in Harari region public health facilities, Eastern Ethiopia. Open J Prevent Med. (2014) 2014:5339. 10.4236/ojpm.2014.47063

[38] Egata G Berhane Y Worku A. Predictors of non-exclusive breastfeeding at 6 months among rural mothers in east Ethiopia: a community-based analytical cross-sectional study. Int Breastfeed J. (2013) 8:8. 10.1186/1746-4358-8-8 23919800 PMC3750393

[39] Mekuria G Edris M. Exclusive breastfeeding and associated factors among mothers in Debre Markos, Northwest Ethiopia: a cross-sectional study. Int Breastfeed J. (2015) 10:1–7. 10.1186/s13006-014-0027-0 25635183 PMC4310186

[40] Mose A Abebe H. Prelacteal feeding practice and its determinant factors among mothers having children less than 6 months of age in Bure district, Northwest Ethiopia: a community-based cross-sectional study. BMJ Open. (2021) 11:e046919. 10.1136/bmjopen-2020-046919 34475152 PMC8413938

[41] Bililign N Kumsa H Mulugeta M Sisay Y. Factors associated with prelacteal feeding in North Eastern Ethiopia: a community based cross-sectional study. Int Breastfeed J. (2016) 11:1–7. 10.1186/s13006-016-0073-x 27190547 PMC4869312

[42] Tariku A Biks GA Wassie MM Gebeyehu A Getie AA. Factors associated with prelacteal feeding in the rural population of northwest Ethiopia: a community cross-sectional study. Int Breastfeed J. (2016) 11:14. 10.1186/s13006-016-0074-9 27231482 PMC4880979

[43] Amele EA Demissie BW Desta KW Woldemariam EB. Prelacteal feeding practice and its associated factors among mothers of children age less than 24 months old in Southern Ethiopia. Ital J Pediat. (2019) 45:15. 10.1186/s13052-019-0604-3 30646943 PMC6334461

[44] Ambaw YL Yirdaw BW Biwota MA Mekuryaw AM Taye BT. Antenatal care follow-up decreases the likelihood of cultural malpractice during childbirth and postpartum among women who gave birth in the last 1-year in Gozamen district, Ethiopia: a community-based cross-sectional study. Arch Public Health. (2022) 80:53. 10.1186/s13690-022-00814-5 35168678 PMC8845281

[45] Temesgen H Negesse A Woyraw W Getaneh T Yigizaw M. Prelacteal feeding and associated factors in Ethiopia: systematic review and meta-analysis. Int Breastfeed J. (2018) 13:1–12. 10.1186/s13006-018-0193-6 30505338 PMC6260692

[46] Kasaye HK Endale ZM Gudayu TW Desta MS. Home delivery among antenatal care booked women in their last pregnancy and associated factors: community-based cross sectional study in Debremarkos town, North West Ethiopia, January 2016. BMC Pregn Childbirth. (2017) 17:1–12. 10.1186/s12884-017-1409-2 28705188 PMC5512956

[47] Regassa LD Tola A Weldesenbet AB Tusa BS. Prevalence and associated factors of home delivery in Eastern Africa: further analysis of data from the recent Demographic and Health Survey data. SAGE Open Med. (2022) 10:20503121221088083. 10.1177/20503121221088083 35342629 PMC8949735

[48] Scott NA Henry EG Kaiser JL Mataka K Rockers PC Fong RM. Factors affecting home delivery among women living in remote areas of rural Zambia: a cross-sectional, mixed-methods analysis. Int J Women's Health. (2018) 10:589–601. 10.2147/IJWH.S169067 30349403 PMC6181475

[49] Tessema KM Mihirete KM Mengesha EW Nigussie AA Wondie AG. The association between male involvement in institutional delivery and women's use of institutional delivery in Debre Tabor town, North West Ethiopia: community based survey. PLoS ONE. (2021) 16:e0249917. 10.1371/journal.pone.0249917 33836011 PMC8034730

[50] Paul PL. The role of male partners in maternal health service utilization: a secondary analysis using 2015–2016 National Family Health Survey (NFHS) data. Midwifery. (2022) 113:103423. 10.1016/j.midw.2022.103423 35870227

[51] Liben ML Yimer NB Feleke FW. Nearly one-in-five mothers avoid colostrum in North Wollo Zone, Ethiopia: an institution-based cross-sectional study. J Nutr Sci. (2021) 10:e100. 10.1017/jns.2021.97 34888038 PMC8634295

[52] Sohail J Khaliq A. Knowledge, attitude and practice of mothers regarding colostrum feeding to newborns in rural pakistan: a cross-sectional study. Khyber Med Univ J. (2017) 9:192–6.31492146

