# Supplementary material for: Prevalence of cultural malpractice during the perinatal period and its determinants among reproductive age women in southwest Ethiopia: A community-based cross-sectional study
Source: Front Public Health. 2023 Mar 17;11:1064583. doi: 10.3389/fpubh.2023.1064583 (PMC10064011; doi:10.3389/fpubh.2023.1064583)
Supplement: Supplementary file 1 [file Table_1.DOCX]

**QUESTIONNAIRE**

**Verbal Informed Consent Form**

Dear participant, Greetings.

My name is ___________________. I am here on behalf of Abinet Tesfaye and his colleagues, who are undertaking a research on “Prevalence of cultural malpractice during the perinatal period and its determinants among reproductive age women in southwest Ethiopia. A community-based cross-sectional study”. You are randomly selected to be part of the study, and I kindly request you to participate in this study which is totally voluntary. The information you provide will help to achieve the study objectives, which are essential to improve maternal and child health through evidence based care.

There will be no harm resulting from this study; however, about 40 minutes of your time will be used for the interview. I assure you that all information collected from you will be confidential. You do not have to answer any question that you don't want to answer and/or you may refuse to answer all of the questions. Besides, you may withdraw from the study at any time and there will be no consequence for it. I appreciate your kindness to be part of the study.

Do you have any questions? Please let me know if anything I have stated is not clear and I will be happy to explain it further to ensure you understand it.

So, are you willing to participate?

If the answer is, **Yes** Continue.

If **No** Thank her and stop.

**Thank you very much!**

*_______________*

Interviewee Code

**Part Ⅰ:** Socio-demographic characteristics of study participants.

| **No** | **Question** | **Response** | **Remark** |
| --- | --- | --- | --- |
| 101 | How old are you? | - 15-19 (1) - 20-24 (2) - 25-29 (3) - 30-34 (4) - 35-39 (5) - 40-44 (6) - 45-49 (7) |  |
| 102 | What is your residence? | - Urban (1) - Rural (2) |  |
| 103 | What is your current marital status? | - Single (1) - Married (2) - Divorced (3) - Widowed (4) |  |
| 104 | What is your educational status? | - No formal education (1) - Primary (2) - Secondary (3) - Tertiary and above (4) |  |
| 105 | What is your occupation? | - House wife (1) - Government employee (2) - Merchant (3) - Other (specify) __________ |  |
| 106 | What is your ethnicity? | - Bench (1) - Kefa (2) - Oromo (3) - Amhara (4) - Other (specify) _____________ |  |
| 107 | What is your religion? | - Orthodox (1) - Protestant (2) - Muslim (3) - Other (specify) __________ |  |

**Part Ⅱ**: Obstetrical characteristics of study participants

| **No** | **Question** | **Response** | **Remark** |
| --- | --- | --- | --- |
| 201 | How many pregnancies have you ever had? | - 1-2 (1) - 3-4 (2) - 5-6 (3) - 7 and above (4) |  |
| 202 | Have you experienced any illness during your recent pregnancy? | - Yes (1) - No (2) |  |
| 203 | If “**Yes**” to question “**202**”, where do you go to receive care? | - Health facility (1) - Traditional birth attendant (2) - Traditional healer (3) - Other (specify) ___________ |  |
| 204 | Do you have ANC follow-up for your recent pregnancy? | - Yes (1) - No (2) |  |
| 205 | Did you feed colostrum to your baby? | - Yes (1) - No (2) |  |

**Part Ⅲ**: cultural malpractices

| **No** | **Question** | **Response** | **Remark** |
| --- | --- | --- | --- |
| 301 | Did you practice any food prohibition during pregnancy? | - Yes (1) - No (2) |  |
| 302 | If “**Yes**” to question “**301**”, which foods did you avoid during pregnancy? | _______________________ |  |
| 303 | Where did you deliver your last child? | - Health facility (1) - Home (2) |  |
| 304 | If your last child was borne at home, who attended the labor? | - Family (1) - Neighbor (2) - Traditional birth attendant (3) - Other (specify) __________ |  |
| 305 | Did you give anything to  drink and/or eat before breast milk within 3 days for your child, after birth? | - Yes (1) - No (2) |  |
| 306 | If “**Yes**” to question “**305**”, what was given to the newborn? | _________________________ |  |
| 307 | What other practices like these (malpractices) do you exercise? | __________________________ |  |
